# Supplementary material for: Chronic Exposure to Paraquat Induces Alpha-Synuclein Pathogenic Modifications in Drosophila
Source: Int J Mol Sci. 2021 Oct 27;22(21):11613. doi: 10.3390/ijms222111613 (PMC8584077; doi:10.3390/ijms222111613)
Supplement: Supplementary file 1 [file ijms-22-11613-s001.zip › Supplemental legends.pdf]

Supplemental Figure S1. Age dependent αSyn resistance to proteinase-K in αSynA53T-expressing flies

Heads form 10-, 20- and 50-day-old flies were digested with increasing concentrations of proteinase-K (PK, from 0 to 2 µg/mL) and analyzed by Western blot with an anti-αSyn (MJFR1) antibody.

Supplemental Figure S2. Age-dependent αSyn solubility in αSynA53T flies

Protein extracts from heads of 1-, 10-, 20-, 30-, 40- or 50-day-old flies expressing αSynA53T were obtained using different lysis buffers ($LB_1^{0.5\%\ NP40}$, $LB_2^{1\%\ NP40\ +\ DTT}$ or $LB3^{urea/thiourea}$) and analyzed by Western blot with the anti-αSyn (MJFR1) antibody.

Supplemental Figure S3. Effect of chronic exposure of PQ on αSyn detectability using LB2 lysis buffer

Quantification of αSyn detection by Western blot (antibody MJFR1) analyses from control (untreated) or PQ exposed (+ PQ) flies expressing αSynWT (A) or αSynA53T (B). Protein extracts were obtained from fly heads (n=100) homogenized using a lysis buffer 2 ($LB_2^{1\%\ NP40\ +\ DTT}$). The optical density of each sample was measured and normalized using a β-tubulin run on the same gel. The graphs are composites of 10 independent experiments (n= 5 for αSynWT and n= 5 for αSynA53T). Data are shown as mean with SD and the dotted line represents the quantity of αSyn detected in untreated flies.
