# Supplementary figures and images for: Chronic Exposure to Paraquat Induces Alpha-Synuclein Pathogenic Modifications in Drosophila
Source: Int J Mol Sci. 2021 Oct 27;22(21):11613. doi: 10.3390/ijms222111613 (PMC8584077; doi:10.3390/ijms222111613)

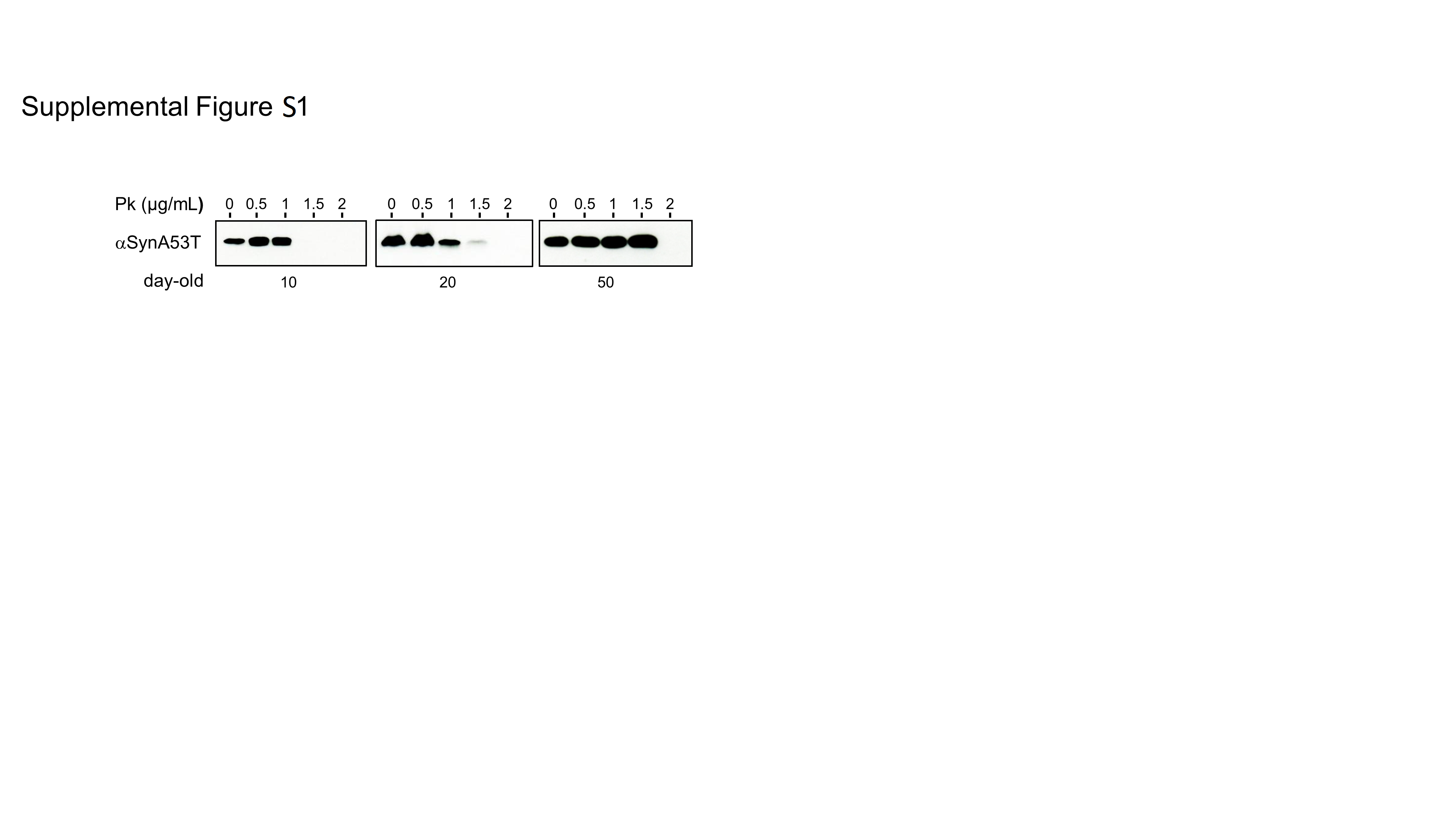

Supplement: Supplementary file 1 [file ijms-22-11613-s001.zip › Supplemental Figure1.TIF]

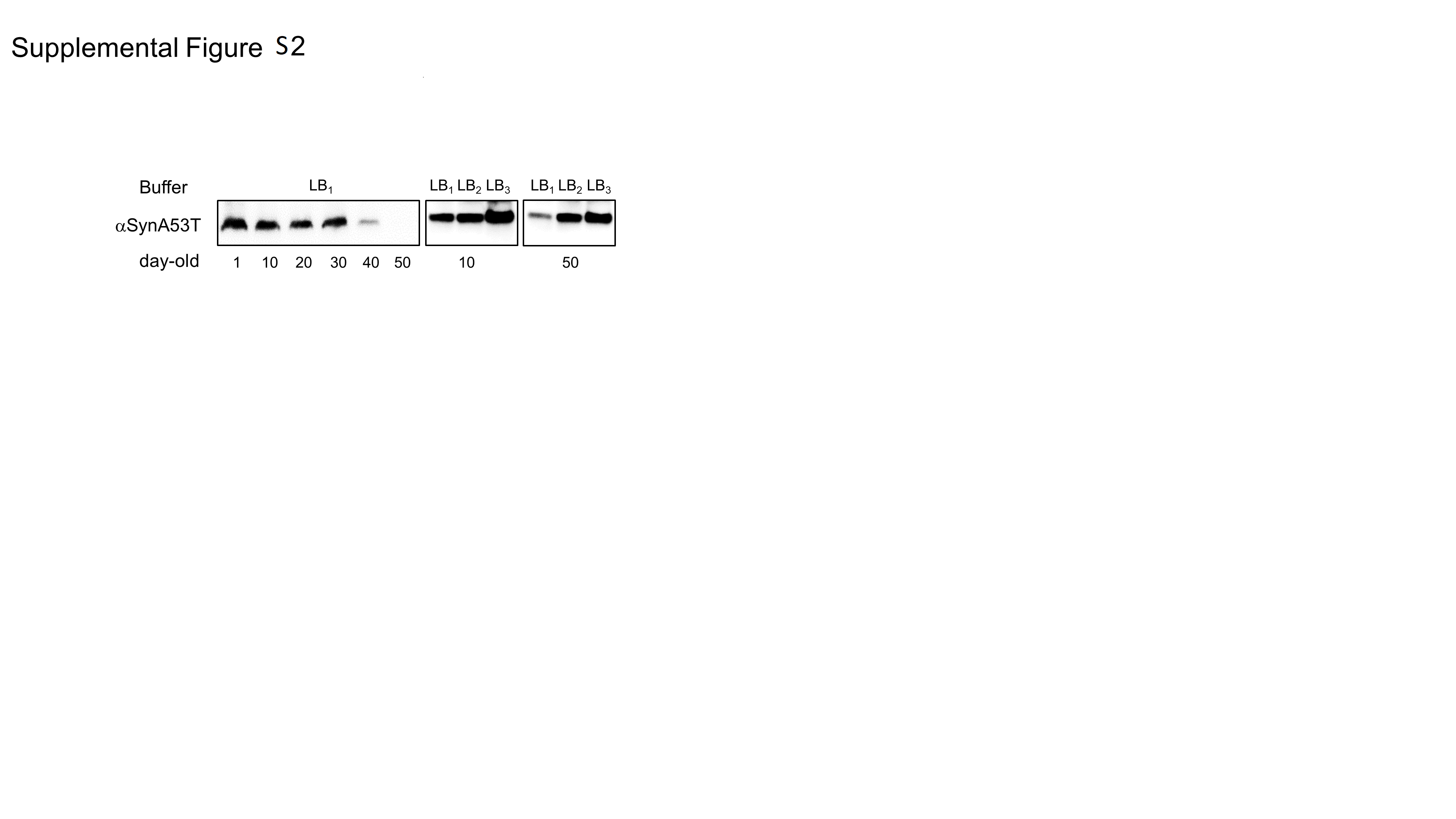

Supplement: Supplementary file 1 [file ijms-22-11613-s001.zip › Supplemental Figure2.TIF]

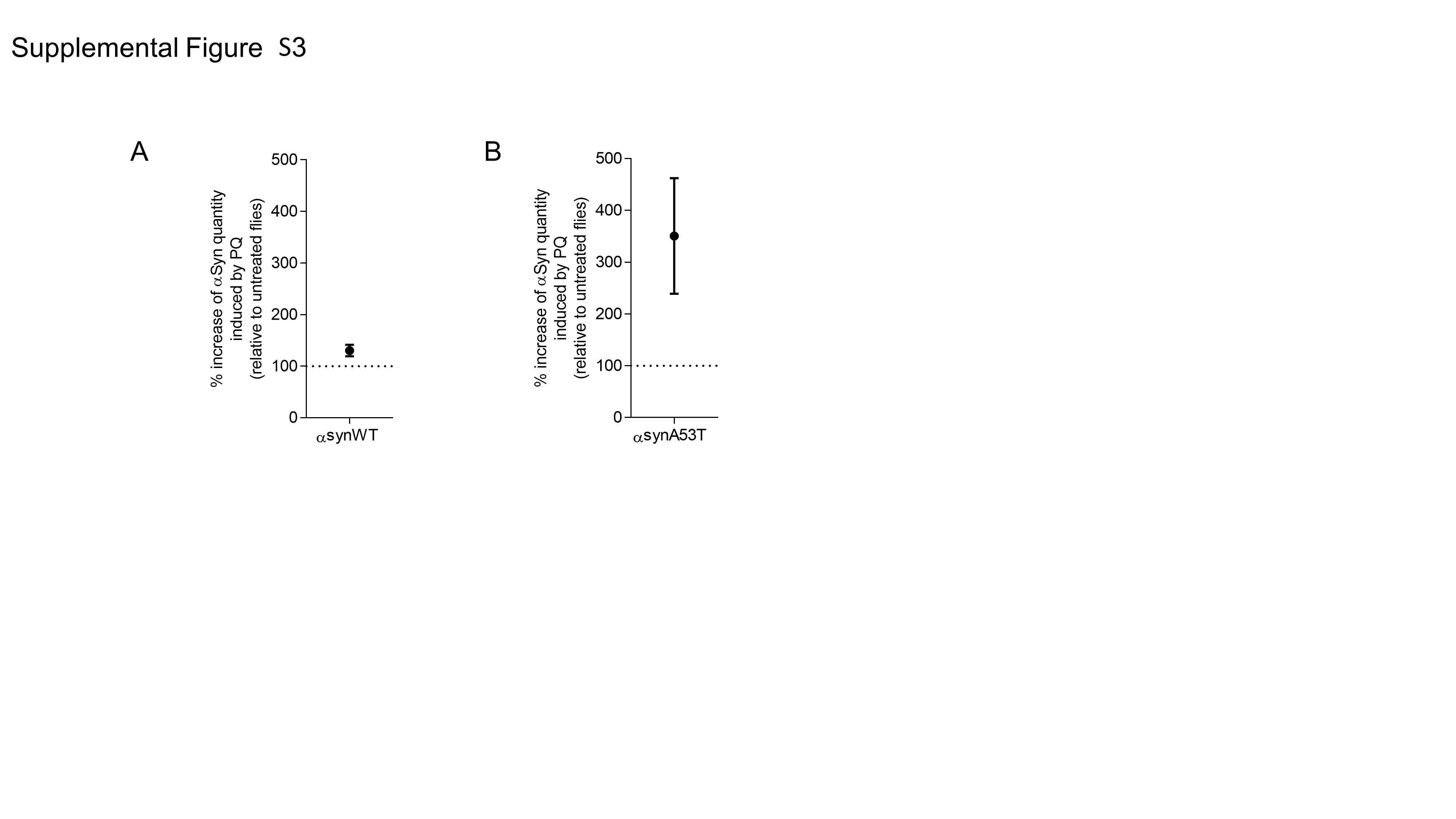

Supplement: Supplementary file 1 [file ijms-22-11613-s001.zip › Supplemental Figure3.TIF]

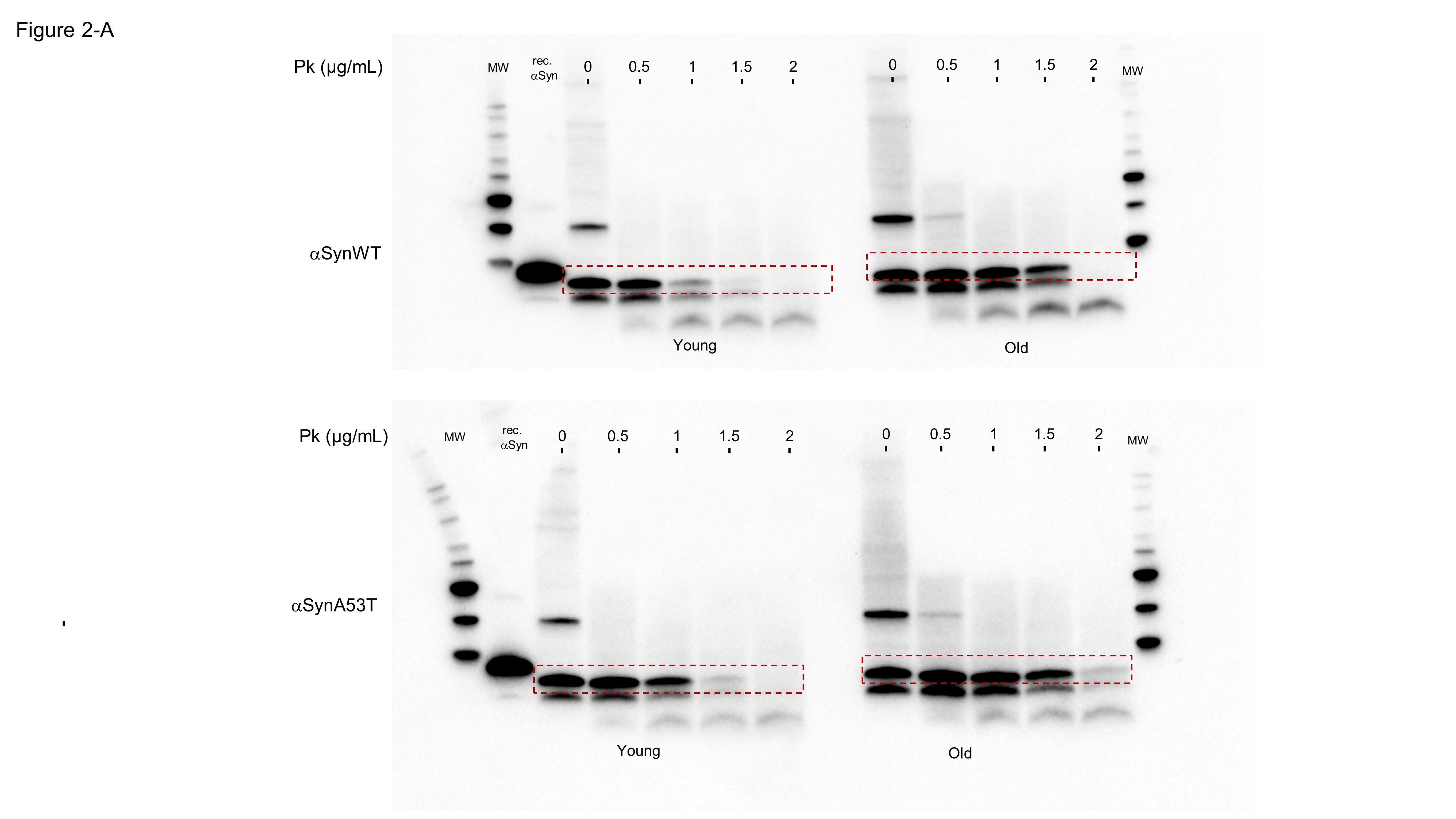

Supplement: Supplementary file 1 [file ijms-22-11613-s001.zip › Supplemental raw data/RawData_Figure2A.TIF]

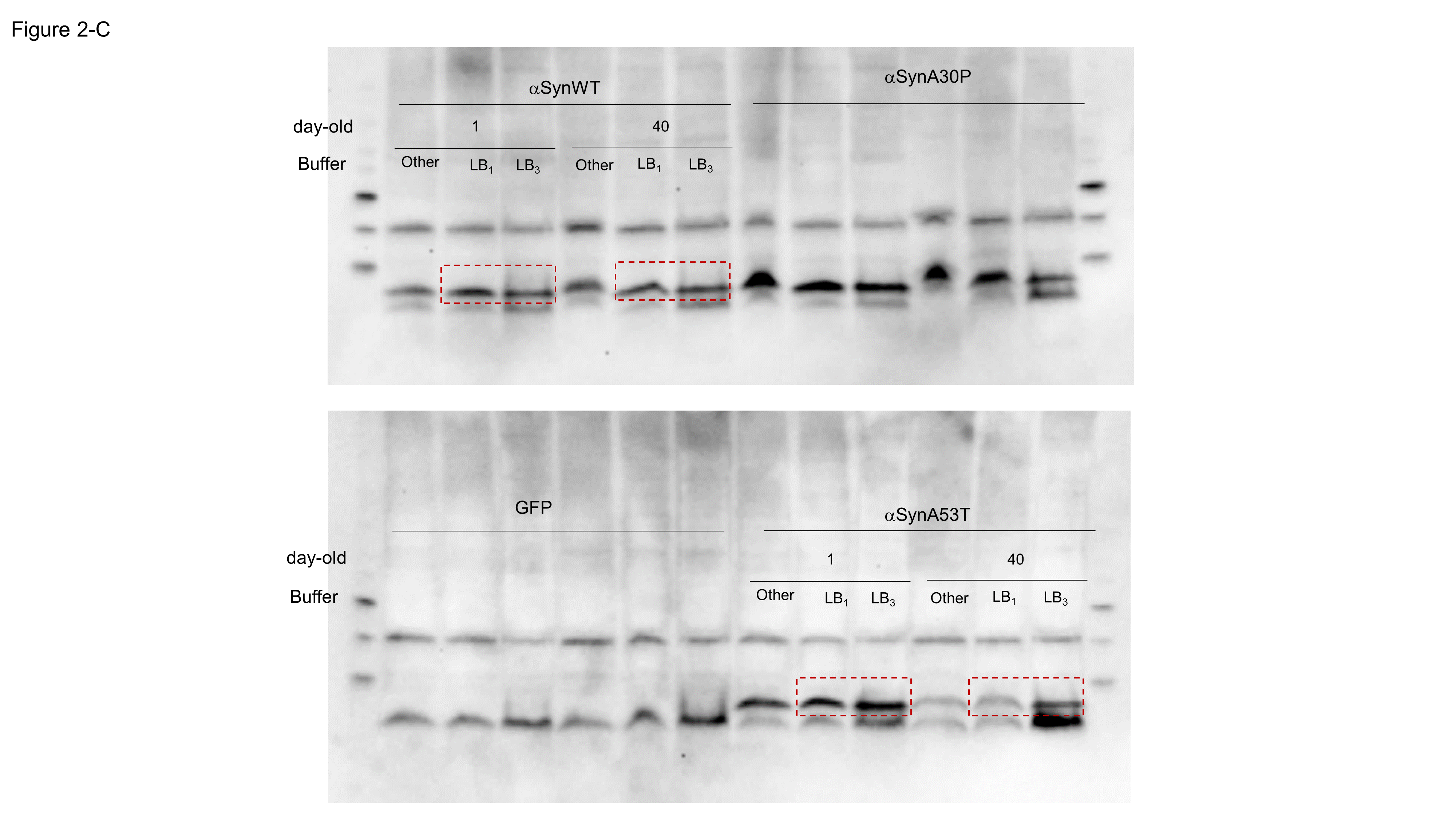

Supplement: Supplementary file 1 [file ijms-22-11613-s001.zip › Supplemental raw data/Rawdata_Figure2C.TIF]

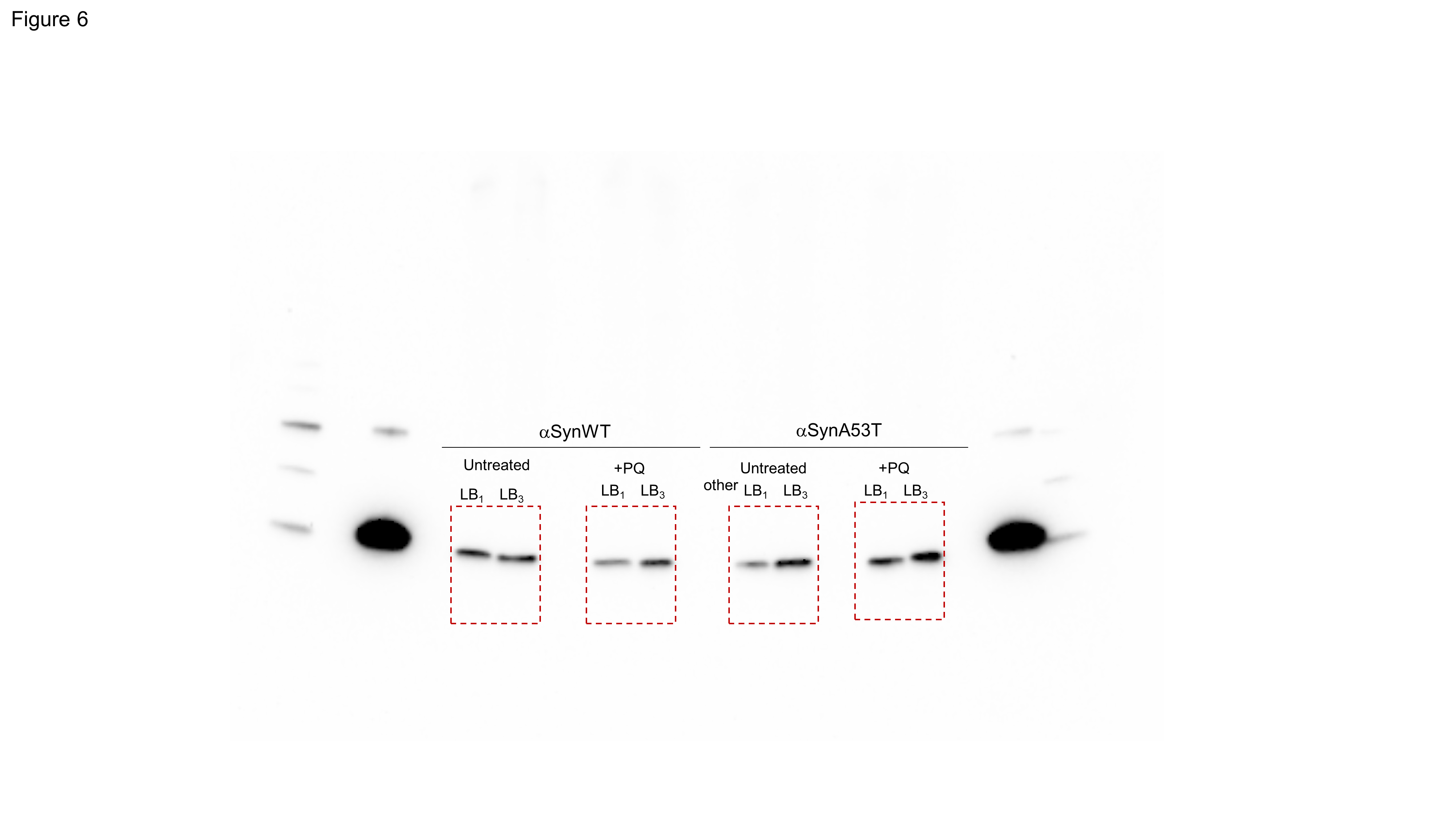

Supplement: Supplementary file 1 [file ijms-22-11613-s001.zip › Supplemental raw data/RawData_Figure6.TIF]

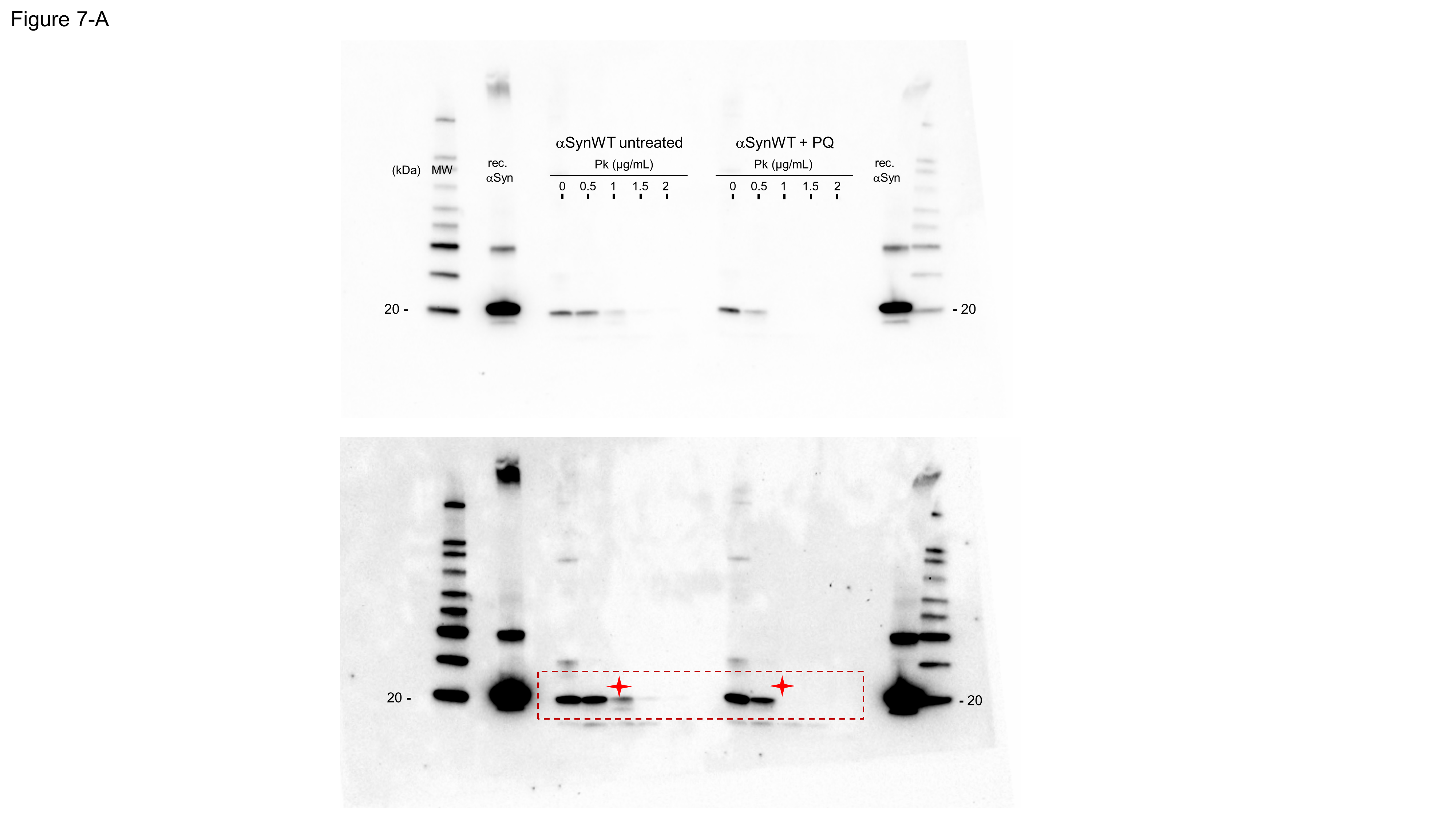

Supplement: Supplementary file 1 [file ijms-22-11613-s001.zip › Supplemental raw data/RawData_Figure7A.TIF]

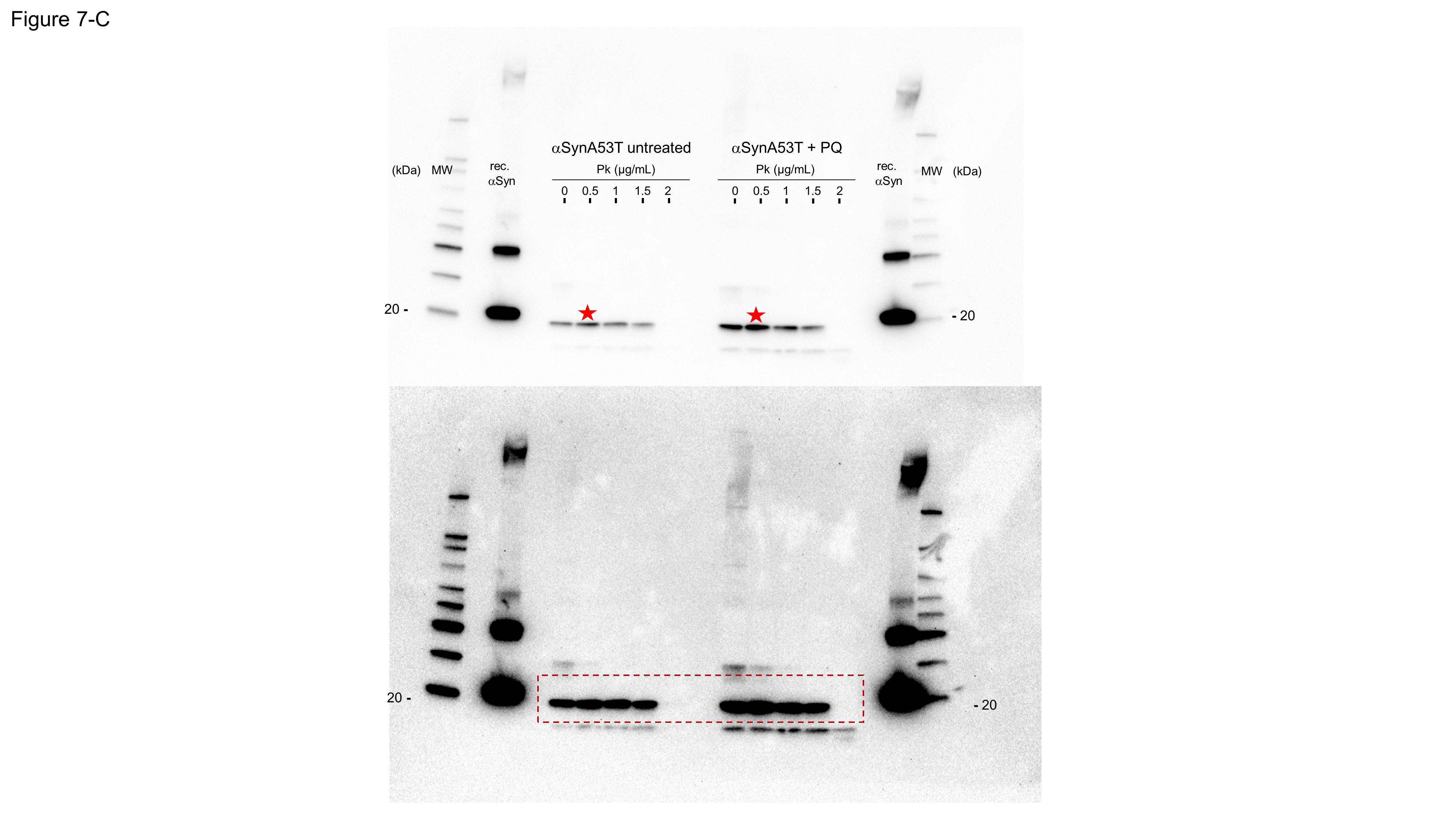

Supplement: Supplementary file 1 [file ijms-22-11613-s001.zip › Supplemental raw data/RawData_Figure7C.TIF]

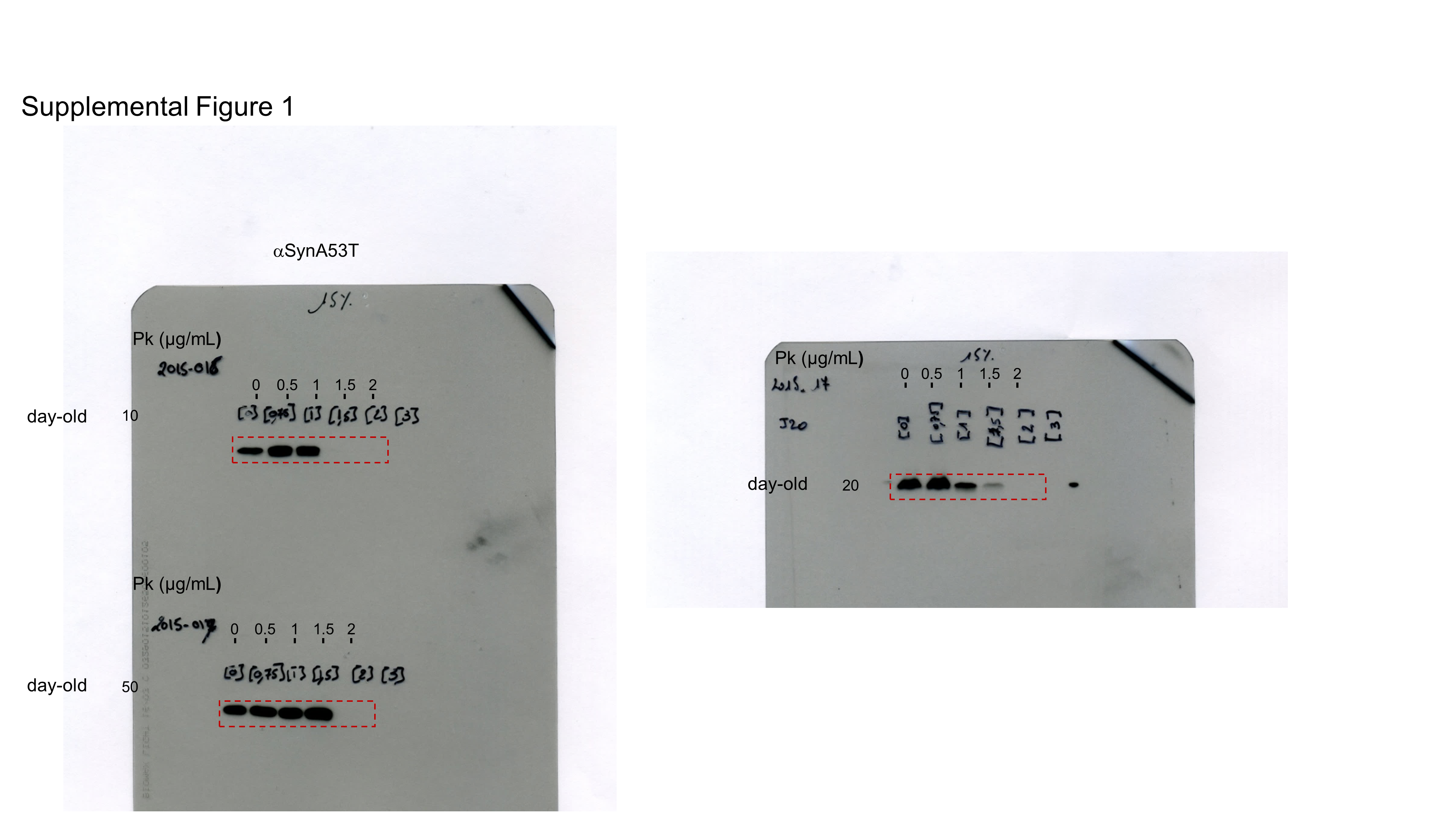

Supplement: Supplementary file 1 [file ijms-22-11613-s001.zip › Supplemental raw data/RawData_supplemental_Figure1.TIF]

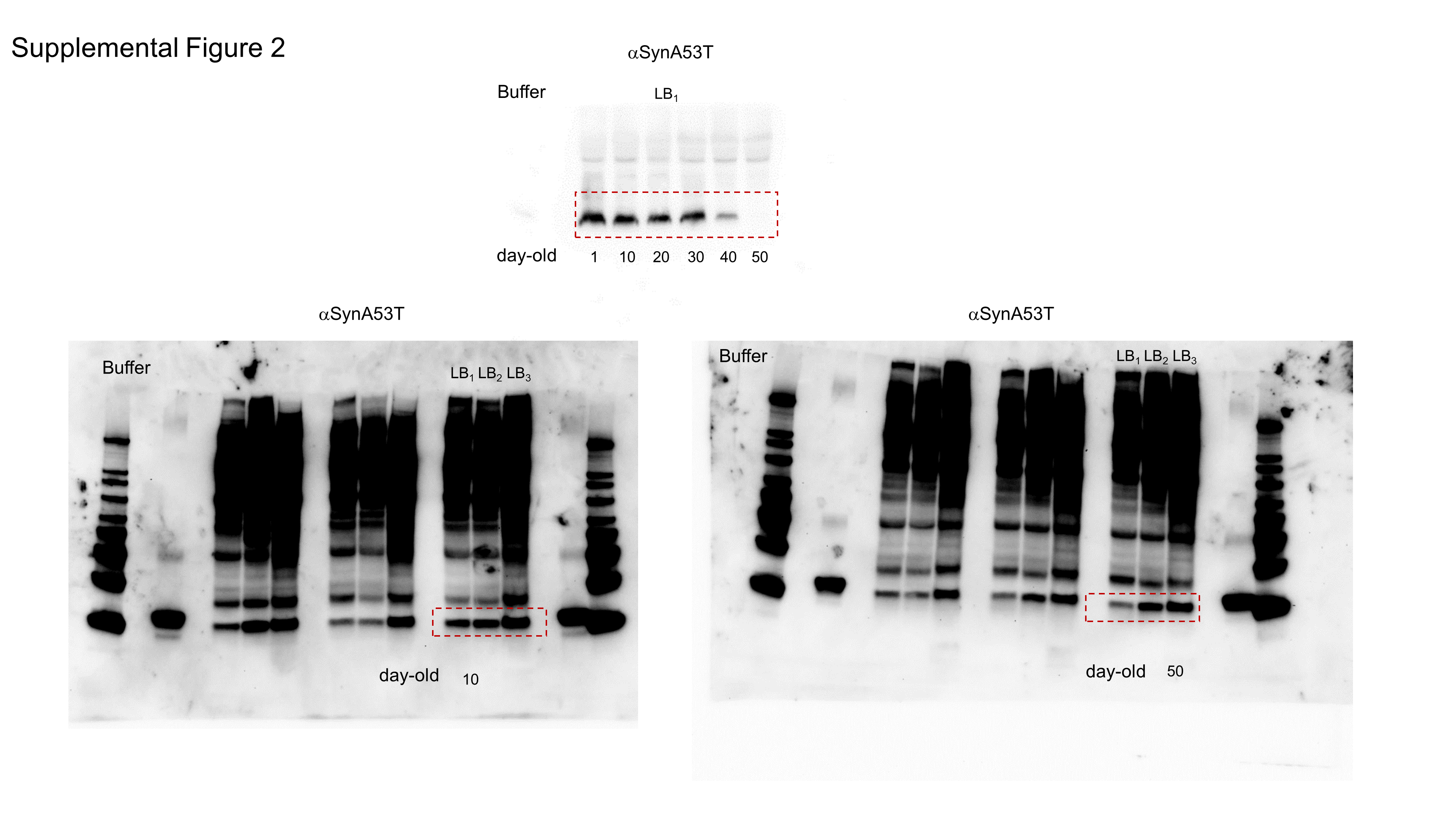

Supplement: Supplementary file 1 [file ijms-22-11613-s001.zip › Supplemental raw data/RawData_supplemental_Figure2.TIF]
